# Pregnancy and Delivery After Solid Organ and Uterus Transplantation: A Review

**DOI:** 10.3390/jcm14145138

**Published:** 2025-07-19

**Authors:** Iori Kisu, Mitsutoshi Yamada, Satoru Ikenoue, Wataru Yamagami

**Affiliations:** Department of Obstetrics and Gynecology, Keio University School of Medicine, Tokyo 160-8582, Japan; mitsutoshi.yamada@gmail.com (M.Y.); ikenouesatoru@me.com (S.I.); gami.z8@keio.jp (W.Y.)

**Keywords:** uterus transplantation, uterine factor infertility, organ transplantation, pregnancy, Mayer–Rokitansky–Küster–Hauser syndrome

## Abstract

In recent years, advances in organ transplantation medicine have led to an increase in pregnancies and births following transplantation. Pregnancy after organ transplantation is considered high-risk, and its impact on both the recipient and the child must be carefully evaluated. In this review, we summarize the current landscape of pregnancy and childbirth after organ transplantation, with a particular focus on uterus transplantation (UTx). Traditionally, organ transplants have involved vital organs; however, UTx, developed for women with absolute uterine factor infertility, represents a novel approach. Although the number of births following UTx remains limited, it is expected to grow due to the international expansion of this procedure. Importantly, the concept of pregnancy and delivery following UTx is fundamentally different from that of other organ transplants. UTx is a life-enhancing, non-vital, and temporary transplant uniquely intended to enable the creation of new life. Pregnancy after UTx carries specific risks such as a higher incidence of miscarriage, preterm birth, hypertensive disorders of pregnancy, and gestational diabetes. All deliveries are performed via cesarean section, and conception is typically allowed after a relatively short period following transplantation, given the temporary nature of the graft and the goal to minimize recipient burden, with generally good neonatal outcomes. As pregnancies after both solid organ transplantation and UTx continue to rise worldwide, the development of standardized, organ-specific perinatal management strategies, particularly for UTx, is essential. Multidisciplinary collaboration will be critical to supporting these high-risk pregnancies and ensuring the best possible maternal and neonatal outcomes.

## 1. Introduction

In recent years, advances in organ transplantation medicine have led to the global expansion of transplant procedures. Improved quality of life and extended life expectancy among patients receiving immunosuppressive therapy have enabled many young women to consider childbearing. According to the Transplant Pregnancy Registry International (TPR), more than 3000 pregnancies occurred in transplant recipients as of 2018 [1], and this number is expected to continue rising. However, pregnancies following organ transplantation carry various risks and complications, including infections related to immunosuppressive agents, graft rejection, long-term functional changes in the transplanted organ, and chronic drug toxicity. Additionally, these pregnancies are classified as high-risk, with a significantly higher incidence of hypertensive disorders, gestational diabetes, preterm delivery, and fetal growth restriction compared to the general population [2]. Therefore, several factors remain under consideration regarding pregnancy after organ transplantation, including physiological changes in the mother during pregnancy, immunosuppressive therapy and rejection during pregnancy, pregnancy complications, delivery methods, and effects on the child.

Uterus transplantation (UTx) represents a novel option for women with absolute uterine factor infertility to achieve childbirth, following the first successful delivery in 2014 [3]. To date, more than 90 UTx procedures have been performed worldwide, resulting in the birth of over 45 healthy infants [4,5]. Although the total number of births after UTx remains limited, it is expected to increase as the procedure gains wider international adoption. Unlike conventional organ transplants, UTx is neither life-saving nor permanent, making it distinct in purpose and nature. Numerous complications associated with pregnancies after UTx have been reported, highlighting the unique challenges of this procedure. In this review, we summarize the current status of pregnancy and delivery following organ transplantation, with a particular focus on UTx.

## 2. Physiological Changes Associated with Pregnancy

Pregnancy induces substantial anatomical and physiological changes in the mother, and understanding these changes is essential for recognizing the pathogenesis of various complications associated with post-transplant pregnancy and for implementing appropriate perinatal management strategies. Maternal body weight increases by approximately 20% during pregnancy, and circulating blood volume expands to support the fetoplacental circulation. This increase in blood volume peaks at around 40% between 28 and 32 weeks of gestation and is maintained until delivery [6]. Cardiac output rises, peripheral vascular resistance decreases due to elevated progesterone levels, and blood pressure remains stable or slightly decreases. In women who are receiving immunosuppressive therapy, such as after solid organ or uterus transplantation, particular attention is required to manage the reduction in immunosuppressant blood concentrations resulting from the increased circulating volume.

In the respiratory system, the diaphragm elevates due to uterine enlargement, leading to decreased residual volume and functional residual capacity, while tidal volume and minute ventilation increase. In the urinary tract, both the pressure exerted by the growing uterus and the smooth muscle relaxation caused by progesterone contribute to ureteral dilation and renal pelvic enlargement. Furthermore, increased intra-abdominal pressure from bladder compression can lead to vesicoureteral reflux, predisposing patients to urinary tract infections. The rise in renal plasma flow lowers serum levels of creatinine, urea nitrogen, and uric acid, while increasing creatinine clearance.

The sex steroid hormones estrogen and progesterone are secreted by the corpus luteum until approximately 10–12 weeks of gestation, after which the placenta becomes the primary source. These hormones increase insulin resistance, leading to hyperglycemia and hyperinsulinemia and thereby enhancing glucose availability for the fetus [7]. Notably, pregnancy is associated with elevated levels of coagulation factors and fibrinogen, resulting in a hypercoagulable state that increases the risk of thrombus formation.

## 3. Time Interval to Pregnancy After Organ Transplantation

In the Transplant Pregnancy Registry International (TPR) 2018 annual report, 1155, 310, 101, 38, 6, and 2 recipients became pregnant following a transplantation of the kidney, liver, heart, lung, pancreas, and small intestine, respectively [1]. Therefore, with more than 45 reported cases, UTx now ranks as the fourth most common type of organ transplant associated with pregnancy [4,5]. The conditions under which pregnancy is permitted after organ transplantation vary depending on the organ type. Among the most essential criteria are stable graft function, good general health of the recipient, an appropriate interval since transplantation, and the resolution or stabilization of rejection episodes. In life-saving organ transplants, the recipient’s ability to tolerate the significant physiological changes of pregnancy and childbirth must be carefully assessed. Accordingly, pregnancy is generally not recommended until organ viability has been confirmed. Because of these concerns, pregnancy within one to two years of transplantation is not advised following conventional life-saving organ transplants [2].

In the context of UTx, the concept of timing pregnancy after transplantation differs from that of life-saving transplants, which require long-term graft function for survival. UTx is a life-enhancing, non-vital, and temporary procedure specifically intended to enable pregnancy. When the first clinical trial of UTx began in Sweden in 2012 [8], the Swedish protocol initially included a 12–18-month waiting period for ET, modeled after recommendations for other solid organ transplant recipients. However, in order to reduce the risks and long-term effects of immunosuppressive therapy, recent approaches have aimed to shorten the recipient graft time, defined as the period between UTx and graft hysterectomy [9,10]. A group in Dallas reported the first live birth after UTx in the United States using a 6-month interval from transplant to ET, followed by immediate hysterectomy after delivery to further minimize graft exposure. In their cohort study, 18 children were successfully delivered using a protocol involving a 3–8-month interval to ET after UTx [11,12,13,14].

Because UTx recipients are typically young and otherwise healthy, and because the transplant is temporary and its goal is to achieve early pregnancy and subsequent delivery, new criteria for timing pregnancy after UTx should be based on balancing graft function and minimizing risk to the recipient and fetus. Additionally, when considering a second pregnancy, embryo transfer should be avoided for at least six months following cesarean section. Evidence indicates an increased risk of uterine rupture when the interval between deliveries is 18–24 months or less [15,16], and a higher risk of maternal morbidity and need for blood transfusion has been observed with interpregnancy intervals shorter than six months [17,18], as reported in the Obstetric Care Consensus by the American College of Obstetricians and Gynecologists and the Society for Maternal-Fetal Medicine [19].

## 4. Immunosuppressive Therapy for Rejection During Pregnancy

In the context of immunosuppressive therapy during pregnancy, mycophenolate mofetil is known to be teratogenic. It is therefore recommended that this medication be discontinued at least six weeks prior to conception or replaced with azathioprine, which carries a lower risk of fetal abnormalities [20]. Notably, the UTx protocol adopted by the Dallas group avoids the use of MMF altogether and instead utilizes azathioprine, which has shown both low teratogenicity and efficacy in preventing acute cellular rejection, with a favorable safety profile for both mother and fetus [14]. Regarding hypertension, a common complication following kidney transplantation, the renin–angiotensin–aldosterone system plays a crucial role in fetal kidney development. Consequently, angiotensin-converting enzyme inhibitors and angiotensin II receptor blockers are contraindicated during pregnancy, as they have been associated with fetal growth restriction, oligohydramnios, congenital anomalies, neonatal renal failure, and neonatal death. To manage hypertension safely during pregnancy, patients are typically transitioned from these agents to medications confirmed to be safe, such as methyldopa [21].

Rejection episodes during pregnancy must be managed cautiously, with consideration for both maternal and fetal outcomes. Reported rates of rejection during pregnancy vary by organ type: kidneys, 0–15% [1,2,21,22]; liver, 1–15% [1,23,24,25]; pancreas, 3–14% [26,27]; heart, 7–12% [1,27,28]; lungs, 10% [1,29]; and small intestine, 0% [30,31,32,33,34] (Table 1). In the case of UTx, a research group in Dallas reported rejection in 2 of 14 pregnancies (14%) [14], and a recent review found rejection in 4 of 50 pregnancies (12.5%) [35]. In all UTx cases, rejection was successfully treated with dosage adjustments of immunosuppressants and the administration of high-dose corticosteroids.

However, high-dose corticosteroid use in late preterm pregnancy may suppress neonatal adrenal function [36,37], necessitating careful monitoring near delivery. Additionally, corticosteroid use in early pregnancy has been linked to an increased risk of cleft lip and palate in infants [38,39,40]. Agents such as anti-human thymocyte rabbit immunoglobulin, rituximab, and basiliximab are not part of standard maintenance immunosuppression regimens. Their use is generally limited to induction therapy or, when necessary, in the management of steroid-resistant acute rejection. While no congenital malformations have been reported in the limited number of pregnancies exposed to rituximab [41], the drug crosses the placental barrier, and reductions in neonatal peripheral lymphocyte counts have been observed. Therefore, its use during pregnancy should be approached with caution.

## 5. Complications During Pregnancy After Organ Transplantation

Pregnancies following organ transplantation are considered high-risk across all graft types when compared with pregnancies in the general population [42,43,44,45,46]. Specifically, the risks of miscarriage, preterm birth, low birth weight, pregnancy-induced hypertension, intrauterine growth restriction, gestational diabetes, and urinary tract infections are significantly elevated in transplant recipients, as shown in Table 1. Therefore, meticulous clinical management is required throughout pregnancy in this population.

Pregnancies following UTx are also classified as high-risk and present several unique challenges not typically observed in recipients of other SOTs. While complications such as miscarriage, preterm birth, hypertensive disorders of pregnancy, and gestational diabetes are common to both SOT and UTx pregnancies, the underlying etiologies and incidence patterns in UTx may differ. Recent systematic reviews and cohort studies report miscarriage rates ranging from 19% to 36% and preterm birth rates (<37 weeks’ gestation) ranging from 35% to 75% after UTx [14,35,47,48].

The risk of miscarriage is particularly notable in UTx pregnancies. Multiple early pregnancy losses have been reported, and these may be linked to impaired uterine vascularization or venous outflow obstruction, mechanisms unique to the UTx setting [49,50]. However, these remain speculative hypotheses, and the exact etiology of miscarriage in UTx is not fully understood. Further research is warranted to clarify the pathophysiology of pregnancy loss, as well as the development of placenta previa, which appears to occur more frequently in pregnancies after UTx. Preterm labor and preterm premature rupture of membranes have been reported in 10–47% of UTx pregnancies [35,47]. Low birth weight (<2500 g) was observed in approximately 43% of neonates born after UTx [35]. However, current evidence suggests that this low birth weight is primarily correlated with earlier gestational age at delivery rather than fetal growth restriction. These elevated rates are likely multifactorial, influenced by provider-initiated preterm delivery, immunosuppressant exposure, and suboptimal uterine perfusion.

Hypertensive disorders of pregnancy, including gestational hypertension and preeclampsia, have been observed in approximately 12–26% of UTx pregnancies [14,35,47,48,51]. Gestational diabetes has been reported in 5–12% of cases [14,35,47,48,51], with rates similar to those in other SOT populations. Although cervical insufficiency and early cervical shortening are typically used to assess the risk of preterm birth, their predictive value in UTx is limited. A recent sub-analysis by Johannesson et al. found that cervical length did not correlate with preterm birth in this population, while cervical inflammation and episodes of graft rejection were more predictive [13]. Placenta previa has been reported in 5–13% of UTx pregnancies [13,35,48], a markedly higher incidence than the baseline rate of 4 per 1000 pregnancies in the general population [52]. The pathogenesis of placenta previa in the UTx setting remains unclear. While many complications observed in UTx pregnancies mirror those seen in other SOT recipients, the distinct anatomical, immunologic, and surgical features of UTx create unique perinatal risks. Thus, close monitoring and individualized management strategies are essential to optimize maternal and neonatal outcomes.

## 6. Delivery Method in Pregnancy After Organ Transplantation

In recipients of SOTs other than the uterus, vaginal delivery is generally considered feasible. However, the rate of cesarean delivery remains substantially higher than that in the general obstetric population (Table 1) [1,23,24,25,26,27,30,31,32,33,34,53,54,55,56,57,58,59]. In contrast, all reported deliveries following UTx have been performed via cesarean section. This practice is primarily attributed to fibrotic stenosis at the vaginal anastomosis site, which may obstruct vaginal delivery, and to the uncertainty surrounding contraction patterns in a transplanted uterus [35].

A systematic review by Brännström et al [47]., which included 40 live births after UTx, reported that 100% of deliveries were performed by cesarean section. Of these, 21 births (52.5%) were planned cesarean deliveries and 19 births (47.5%) were emergency cesarean deliveries. Among the planned cesarean sections, 11 were performed before 37 weeks’ gestation, and 10 were conducted at or beyond 37 weeks. The overall rate of preterm birth (<37 weeks) was 28 out of 40 cases (70.0%) [35]. Emergency indications included fetal heart rate abnormalities, labor arrest following premature rupture of membranes, and hypertensive disorders of pregnancy. In early clinical protocols, planned cesarean deliveries were frequently scheduled before 37 weeks’ gestation, contributing to higher rates of preterm birth and low birth weight. However, recent trends have shifted toward scheduling cesarean sections after 37 weeks to improve neonatal outcomes [14,60].

## 7. Neonatal and Infant Outcomes After Delivery in Organ Transplantation

Pregnancies following SOT are associated with a distinct neonatal risk profile, most notably an increased incidence of preterm delivery and low birth weight [1]. For example, emergent cesarean deliveries, more common in kidney and liver transplant recipients, are associated with lower birth weight, earlier gestational age, and a higher frequency of neonatal intensive care unit (NICU) admissions [59]. Nevertheless, the incidence of congenital malformations among infants born to transplant recipients is comparable to that observed in the general population. Large registry-based studies have shown no increased risk of structural anomalies in neonates exposed in utero to commonly used immunosuppressive agents, including calcineurin inhibitors (cyclosporine and tacrolimus), azathioprine, and corticosteroids [1].

Postnatal developmental outcomes in these infants are generally reassuring. Most children born to transplant recipients exhibit normal growth and neurodevelopment, with no evidence of increased risk for cognitive delays, immunodeficiencies, or chronic health conditions during early childhood. Although some neonates may present with transient renal impairment or mild immunosuppression due to in utero exposure to immunosuppressive therapy, these conditions typically resolve without long-term consequences [61].

Neonatal outcomes following UTx have been increasingly documented over the past decade. Birth weights below the 10th and 20th population percentiles occurred in 20.0% (8/40) and 12.5% (5/40) of infants, respectively, rates that exceed the background prevalence of 5.7%, underscoring a persistent risk of fetal growth restriction [35,62]. No neonates delivered after UTx had Apgar scores below five at five minutes [35]; median Apgar scores were eight at one minute and nine at five minutes, both within the reassuring range [63,64,65].

Respiratory distress syndrome (RDS) occurred in 14 of 40 neonates (35%), of whom 13 required continuous positive airway pressure. Most NICU admissions were brief, with a median stay of 2.5 days (range, 1–79 days), and prolonged admissions (>1 week) were limited to three very preterm infants born between 28 and 31 weeks of gestation. Only one minor congenital anomaly, a displaced female urethra, was reported among the initial 40 infants (2.4%), a rate consistent with the expected prevalence of 2–4% in the general population [66]. Similarly, an independent review identified three additional minor anomalies (patent foramen ovale, transient clitoromegaly with anterior urethral displacement, and helical ear indentation), none of which appeared to be related to maternal immunosuppressive exposure [63].

Among the 17 children who completed two years of follow-up, only one was diagnosed with a mild, transient neurodevelopmental delay, which subsequently improved [67]. Overall, physical, neurological, and immunological developmental milestones during the first two years of life were age-appropriate. Importantly, no cases of severe hypoxic-ischemic encephalopathy, meconium aspiration, perinatal mortality, or graft-specific neonatal pathology have been reported. Overall neonatal morbidity in UTx appears to be driven primarily by gestational age at delivery rather than by the transplant procedure itself.

Currently, direct comparisons of neonatal outcomes between UTx and other solid organ transplants are limited by the small number of births following UTx and the differences in clinical context. In particular, many births after UTx have occurred during early implementation phases, where elective preterm delivery was commonly chosen. As a result, the high rates of preterm birth may not solely reflect the underlying biology of UTx pregnancies but also intentional clinical decisions. Further longitudinal studies with larger cohorts will be necessary to accurately assess and compare neonatal outcomes across transplant types. To mitigate the risks of preterm delivery and low birth weight in pregnancies after organ transplantation, a multidisciplinary and individualized approach is essential. This includes careful fetal growth monitoring, tailored timing of delivery based on maternal and graft condition, and appropriate adjustment of immunosuppressive regimens to balance graft protection with fetal development.

## 8. Conclusions

Pregnancy after organ transplantation has evolved from a rare and high-risk consideration to an increasingly common and achievable outcome, owing to advances in surgical techniques, immunosuppressive regimens, and perinatal care. While all pregnancies following SOT are classified as high-risk, due to elevated rates of preterm birth, low birth weight, and maternal complications, UTx represents a fundamentally different paradigm. Unlike conventional transplants, which are life-saving interventions intended to extend survival, UTx is a life-enhancing, non-vital, and temporary procedure designed uniquely to enable childbearing.

As the global number of pregnancies following both SOT and UTx continues to rise, the development of standardized, organ-specific perinatal management strategies becomes increasingly important, particularly for UTx. Multidisciplinary collaboration will be essential to support these high-risk pregnancies and to ensure optimal maternal and neonatal outcomes.

## Figures and Tables

**Table 1:** Maternal and pregnancy outcomes following solid organ transplantation.

		General Population	Kidney	Liver	Pancreas *	Heart	Lung	Small Intestine ^#^	Uterus
Recommended interval from transplantation to conception			>1 year	>2 years	>1 year	>1 year	>2–3 years	>2 years	>3–6 months
Incidence of rejection during pregnancy			0–15%	1–15%	3–14%	7–12%	10%	0%	13–14%
Pregnancy complications	Miscarriage	12–20%	17–18%	16–23%	27%	24%	23–26%	11%	19–36%
	Preterm birth	6–11%	33–52%	23–50%	73–80%	43–51%	58–64%	22%	35–75%
	Low birth weight infant	4–15%	42–55%	23–57%	62–71%	38%	82%	22%	43%
	Pregnancy-induced hypertension	5–10%	24–48%	11–43%	52–64%	27–47%	41–56%	Unknown	14–26%
	Gestational diabetes	10–14%	5–9%	8%	3–15%	6–8%	17–32%	Unknown	5–12%
Cesarean delivery rate		15–25%	51–56%	44–60%	72–73%	40–45%	39–50%	44%	100%

* Included kidney–pancreas transplant cases. ^#^ The number of reported pregnancies after intestinal transplantation is limited.

## Data Availability

All data analyzed in this article are included in the manuscript and are available in a publicly accessible repository. Further inquiries can be directed to the corresponding author.

## Abbreviations

The following abbreviations are used in this manuscript:

TPR	Transplant Pregnancy Registry
UTx	Uterus transplantation
ET	Embryo transfer
SOT	Solid organ transplant
NICU	Neonatal intensive care unit
